# Diagnostic accuracy of magnetic resonance imaging for the detection of pulmonary nodules simulated in a dedicated porcine chest phantom

**DOI:** 10.1371/journal.pone.0244382

**Published:** 2020-12-23

**Authors:** Madeleine Bonert, Moritz Schneider, Olga Solyanik, Katharina Hellbach, David Bondesson, Thomas Gaass, Natalie Thaens, Jens Ricke, Thomas Benkert, Julien Dinkel

**Affiliations:** 1 Department of Radiology, University Hospital, LMU Munich, Munich, Germany; 2 Comprehensive Pneumology Center, German Center for Lung Research, Munich, Germany; 3 MR Applications Predevelopment, Siemens Healthcare GmbH, Erlangen, Germany; National Institutes of Health, UNITED STATES

## Abstract

**Objective:**

CT serves as gold standard for the evaluation of pulmonary nodules. However, CT exposes patients to ionizing radiation, a concern especially in screening scenarios with repeated examinations. Due to recent technological advances, MRI emerges as a potential alternative for lung imaging using 3D steady state free precession and ultra-short echo-time sequences. Therefore, in this study we assessed the performance of three state-of-the-art MRI sequences for the evaluation of pulmonary nodules.

**Methods:**

Lesions of variable sizes were simulated in porcine lungs placed in a dedicated chest phantom mimicking a human thorax, followed by CT and MRI examinations. Two blinded readers evaluated the acquired MR-images locating and measuring every suspect lesion. Using the CT-images as reference, logistic regression was performed to investigate the sensitivity of the tested MRI-sequences for the detection of pulmonary nodules.

**Results:**

For nodules with a diameter of 6 mm, all three sequences achieved high sensitivity values above 0.91. However, the sensitivity dropped for smaller nodules, yielding an average of 0.83 for lesions with 4 mm in diameter and less than 0.69 for lesions with 2 mm in diameter. The positive predictive values ranged between 0.91 and 0.96, indicating a low amount of false positive findings. Furthermore, the size measurements done on the MR-images were subject to a bias ranging from 0.83 mm to -1.77 mm with standard deviations ranging from 1.40 mm to 2.11 mm. There was no statistically significant difference between the three tested sequences.

**Conclusion:**

While showing promising sensitivity values for lesions larger than 4 mm, MRI appears to be not yet suited for lung cancer screening. Nonetheless, the three tested MRI sequences yielded high positive predictive values and accurate size measurements; therefore, MRI could potentially figure as imaging method of the chest in selected follow-up scenarios, e.g. of incidental findings subject to the Fleischner Criteria.

## Introduction

Statistics show that lung cancer not only remains a leading cause of cancer-related deaths [1–3], but that it also resides among the top 10 causes of death in general [4], with tobacco smoking representing the most important risk factor [1, 2, 5]. The fatal character of this disease is reflected by poor survival rates [1, 2], which strongly depend on stage at diagnosis [2, 6]. Unfortunately, a high percentage of patients are only diagnosed in advanced stages. For example, in 2018 in the U.S.A., 61% were diagnosed with a distant stage of lung cancer [6]. This is amongst others due to the fact, that early stages of this burden remain asymptomatic or provoke merely unspecific symptoms such as chronic cough. Therefore, efforts are still made to establish screening protocols for high-risk patients (e.g. smokers with ≥ 30 packyears, aged 55–74 years) [7].

Computed tomography (CT) has been established as the gold standard for the radiological assessment of lung parenchyma and for the evaluation of pulmonary nodules. However, one of the main limitations of this imaging modality is the associated radiation dose and the related potential risk of developing a second malignancy [8, 9]. This aspect is particularly relevant for patients undergoing repeated examinations as e.g. in screening scenarios.

Recent technical advances improved the quality of thoracic magnetic resonance imaging (MRI) and gave rise to a potential alternative to CT, void of ionising radiation. Therefore, MRI has already become an important diagnostic tool for the assessment of cystic fibrosis or for pregnant and paediatric patients [10, 11]. The value of MRI for the detection of pulmonary nodules has been reassessed over the past years in several studies comparing the sensitivities of CT and MRI [12–16]. Those showed the great potential of MRI, but also highlighted its inferiority to CT. Biederer et al. found in 2003 that 3D GRE MRI could detect pulmonary nodules of 4.2 mm diameter with a sensitivity of 88%, whereas spiral CT showed a sensitivity for the same nodule diameter of 96% [15]. Furthermore Cieszanowski et al. demonstrated in 2016 an overall sensitivity of 80.5% for MRI. In addition to that, they accounted for a strong congruence of the measured diameters in MRI and CT [14]. Later—in 2018—this was also observed by Meier-Schroers et al., who conducted a lung cancer screening with both low-dose CT and MRI applying the Lung-RADS-criteria [16, 17]. By the time, some developments in image acquisition technique had addressed one main limitation of MRI, its low spatial resolution. They concluded their study with the outlook that Lung-RADS could possibly be applied in a lung cancer screening with MRI [16]. Recent advances in MRI hardware and pulse sequence design have further uncovered the potential of 3D steady state free precession (SSFP) [18] and 3D ultra-short echo-time (UTE) [19] sequences for lung imaging. Therefore, we conducted this study in order to compare current state-of-the-art MRI sequences for lung imaging, consisting of a SSFP sequence, a UTE and a 3D GRE VIBE sequence, for the evaluation of pulmonary nodules simulated in a dedicated porcine chest phantom with conventional CT as reference. As spatial resolution is the crucial point concerning this topic, we selected MRI sequences optimized by parallel imaging, echo time reduction or spiral undersampling.

## Material and methods

### *Ex vivo* chest phantom

The dedicated chest phantom (artiCHEST®, PROdesign GmbH, Heiligkreuzsteinach, Germany) used in this study, is composed of two double-walled shells made of transparent copolymer. The shells form a thoracic cavity, big enough to hold a porcine heart-lung preparation. A 2–5 cm gap between the inner and outer wall of each shell is filled with water in order to simulate the thoracic wall. To mimic the diaphragm, a water-filled silicone implant is integrated in the caudal part of the artificial chest cavity. Through a sealed outlet in the cranial part of the phantom, a 7.5 mm tracheal tube (Portex®, Smiths Medical, Inc., Minneapolis, Minnesota, USA) is introduced in the porcine trachea.

In total we used 8 freshly excised heart-lung preparations from adult pigs, collected at a local slaughterhouse, where the animals were sacrificed for alimentary reasons (Muenchner Schlachthof Betriebs GmbH, Munich, Germany). Initially, we checked the preparations for any lacerations. If present, we sutured them with surgical wire and examined for leak tightness by inflating the lungs test wise with a resuscitation bag. After injecting the simulated nodules (see below), the lungs were placed inside the phantom, which was closed hermitically afterwards.

An aspirator, connected over additional holes to the inside of the phantom, evacuated the air by continuously applying a pressure level of -20 to -30 hPa to the artificial pleural space. Consequently, the lungs filled with ambient air through the tracheal tube and expanded in a physiological manner.

As no human participation, no anaesthesia, no euthanasia, nor any kind of animal sacrifice were part of this study, the approval of the Institutional Review Board or of the Committee on Animal Research and Ethics wasn’t required.

### Simulation of the lung nodules

**Fig 1:**
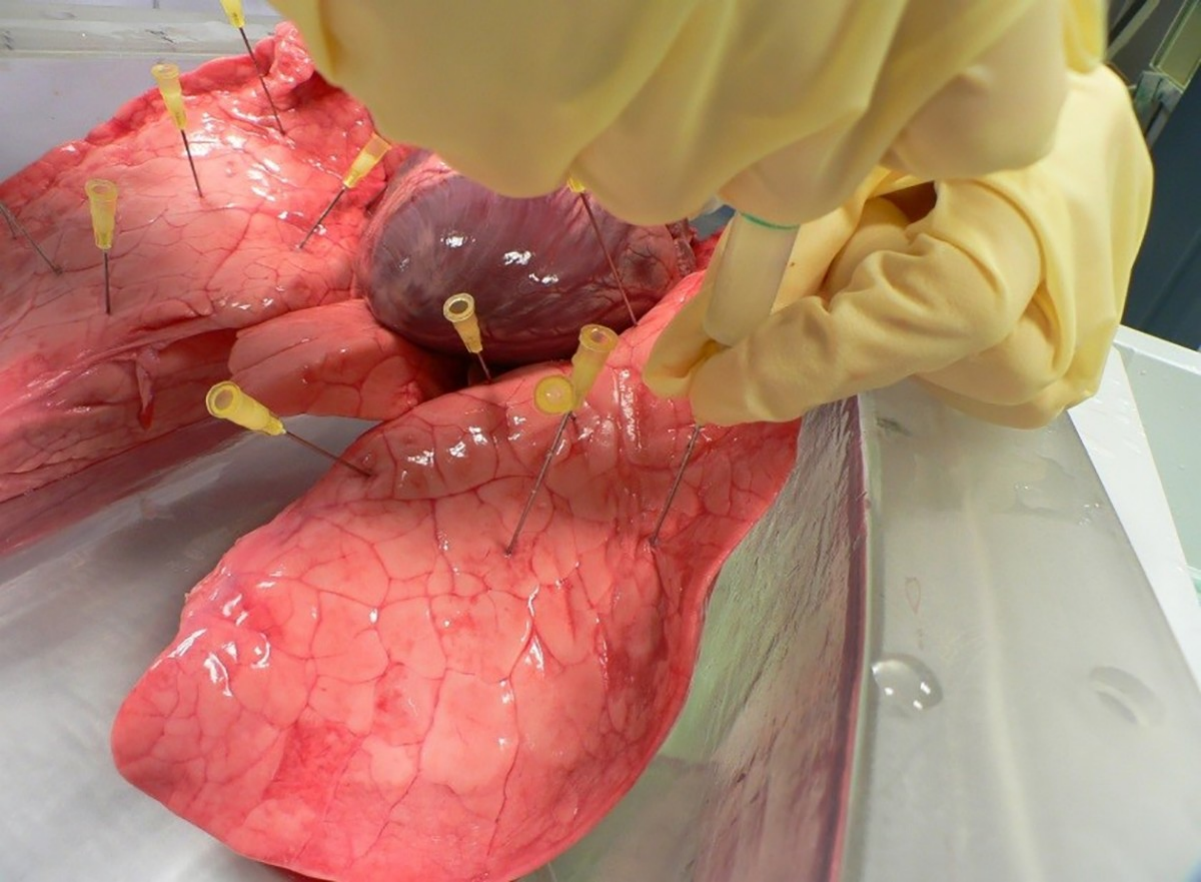
Pulmonary nodules were simulated by injecting 0.3–0.8 mL of our agar-mixture into the lung parenchyma using a 5 mL syringe and a 20 G cannula.

To simulate the T1- and T2-relaxation times of solid pulmonary nodules, we mixed a 3% agar-water solution (Carl Roth GmbH & Co. KG, Karlsruhe, Germany) with prune juice (Haus Rabenhorst O. Lauffs GmbH & Co. KG, Unkel, Germany) in a ratio of 9:1 [20]. As depicted in Fig 1, we injected 0.3–0.8 ml of this mixture into the lung parenchyma at a depth of 3–6 cm using a 5 ml syringe (BD Discardit II®, Becton Dickinson GmbH, Heidelberg, Germany) with a 20 G cannula (Sterican®, B. Braun Melsungen AG, Melsungen, Germany). We disseminated about 16–22 injections per lung, resulting in a total of 280 simulated pulmonary nodules of variable size.

### MRI sequences

Images were acquired with a clinical 1.5 Tesla MR scanner (MAGNETOM Aera, Siemens Healthcare, Forchheim, Germany) using a dedicated body phased array coil.

Our MRI protocol consisted of three sequences: 1. a 3D steady state free precession sequence in coronal orientation (3D SSFP cor), 2. a prototypical 3D gradient-echo sequence with ultra-short echo time of 0.05 ms and stack-of-spirals acquisition scheme [21] in coronal orientation (3D GRE UTE cor) and 3. a 3D gradient-echo sequence with an optimized time of echo of 0.66 ms in transverse orientation (3D GRE tra).

The parameters of each sequence were optimized as summarized in Table 1, in order to enable a breath-hold-acquisition within 18 seconds.

**Table 1:** Imaging parameters of the three MRI sequences.

Parameters	3D SSFP cor (TRUFI)	3D GRE UTE cor (VIBE)	3D GRE tra (VIBE)
**Repetition time/Echo time (ms)**	1.6/0.57	3.39/0.05	2.54/0.66
**Acquisition Matrix**	208 x 161	256 x 256	320 x 157
**Slice thickness (mm)**	2.2	2	2
**Flip angle (°)**	30	5	7
**Number of partitions**	96	96	120
**Acquisition time (s)**	18	18	18
**Field of view (mm^2^)**	450 x 348	500 x 500	400 x 196
**Voxel size (mm^3^)**	2.16 x 2.16 x 2.2	1.95 x 1.95 x 2	1.25 x 1.25 x 2
**Parallel imaging**	Factor 2	Factor 2 with iterative reconstruction SPIRiT	Factor 4 with CAIPIRINHA

**Cor =** coronal orientation; **TRUFI =** true fast imaging with steady-state free precession; **VIBE =** volumetric interpolated breath hold- examination; **Tra =** transverse orientation; **SPIRiT =** iterative self-consistent parallel imaging reconstruction from arbitrary k-space; **CAIPIRINHA = c**ontrolled aliasing in parallel imaging results in higher acceleration

### Computed tomography

Multi-detector computed tomography was performed with a third-generation dual-source CT scanner (SOMATOM Force, Siemens Healthcare, Forchheim, Germany) with parameters set as follows:

Collimation = 0.6 mm; number of slices = 192; gantry rotation time = 0.25–0.5 s; pitch = 0.5–1.2; scan time = 3–10 s; tube current = 540 mA; with 120 kV tube voltage.

The CT images were reconstructed with an iterative algorithm (ADMIRE, Siemens Healthcare, Forchheim, Germany) using a sharp tissue kernel (Br69) at level 3, a slice thickness of 0.75 mm, and a slice increment of 0.6 mm. The images were reconstructed with a matrix of 512 x 512 pixels and with a field of view of 330 mm.

### Image evaluation

Both MR and CT images were reviewed with an oncology software tool (syngo Multimodality Workplace VE36A, Siemens Healthcare, Forchheim, Germany) in order to record the location and to measure the maximum diameter of the artificial nodules.

A reader with 10 years of experience in chest radiology evaluated the CT images of the 8 porcine lungs using the syngo Lung-CAD tool and recording the exact position and diameter of each pulmonary nodule in a dedicated database (Excel 2010, Microsoft Corporation, Redmond, WA, USA). During this meticulous search, some injections were detected in bronchi or pulmonary arteries. In fact, these agar injections take on a very odd, oblong shape within these tubular structures. By consequence, the intrabronchial or intravascular location of these pseudo-nodules was evident on CT, and the senior thoracic radiologist could annotate those findings as false positive. Extra-parenchymal nodules were also considered as artefacts, resulting in a final amount of 254 variably sized pulmonary nodules. This dataset served as our reference standard.

Next, two blinded readers with 2 (Reader A) and 4 (Reader B) years of experience in chest radiology analysed the MR images, searching for suspect lung lesions, marking their location and measuring their diameter using a dedicated software (syngo.via for Oncology, Siemens Healthcare, Forchheim, Germany). The senior thoracic radiologist–who had evaluated the CT images earlier–compared afterwards the MRI findings of both readers with his CT-based dataset, so he could extract intrabronchial and intravascular nodules from the MR tabulation.

For each MR sequence, an individual reading session was performed. Between those three reading sessions, we complied with an interval of 6 weeks.

Finally, the MR assessments were compared to our reference standard. After comparison to the CT images, false-positive findings were categorized as follows: False-positive findings attributed to occlusions in vessels or bronchi (e.g. due to accidental injection of agar-mixture or thrombi) were categorized as “vessel” or “bronchi”, respectively. These false-positives were considered to be artificially caused based on our study design, as they are unlikely to occur in living patients. False-positive findings without a CT correlate were categorized as “other”, representing our true false-positive findings.

### Statistical analysis

The statistical analysis of our raw data was conducted with the free software environment R (R foundation for Statistical Computing, Vienna, Austria).

Logistic regression was used to analyse the probability of nodule detection (sensitivity) as a function of the nodule’s diameter. For comparison, the sensitivity was also analyzed by nodule size intervals.

Furthermore, the positive predictive value (PPV) as an indicator for precision, was calculated for each reader and each MR sequence including all false-positive findings as well as excluding artificial false positives categorized as bronchus or vessel.

Bland-Altman analysis was performed to compare the measured diameters sized in CT and in MR images.

Additionally, we performed an unweighted Kappa test to evaluate the interrater reliability between Reader A and Reader B. An unweighted Kappa between 0.61 and 0.80 was rated as substantial and a value between 0.41 and 0.60 was rated as moderate [22].

## Results

**Fig 2:**
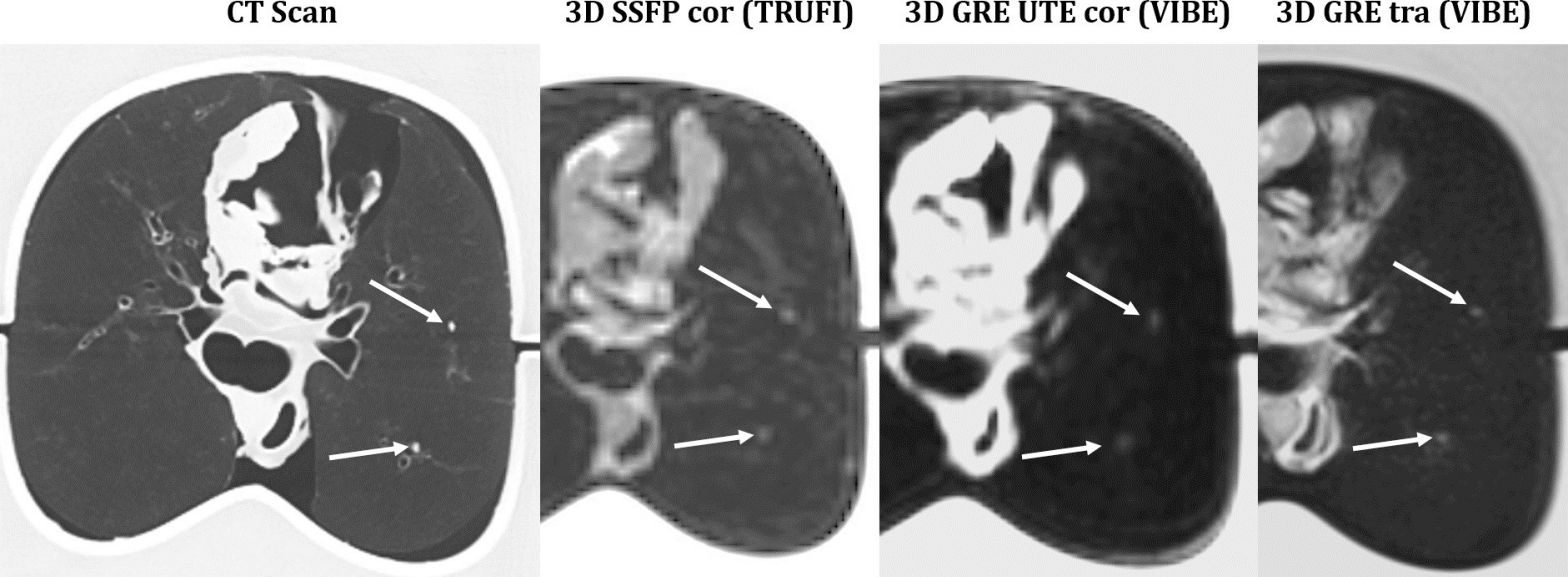
CT-MRI comparison of 2 nodules with a diameter of 2mm.

Comparing MR to CT images (cf. Fig 2), there is still an apparent difference in image quality: smallest alterations are depicted by CT, whereas MR images don’t portray the lung parenchyma and appear unsharp, based on its minor spatial resolution. From the time, when Paul Lauterbur published his theory on magnetic resonance-based imaging in March 1973, spatial resolution has been the Achilles’ heel of MRI. Ever since, all studies comparing MRI and CT for the detection of pulmonary lesions have proven the inferiority of MRI, limited by its spatial resolution. In fact, a high-resolution MR image takes a long acquisition time, conflicting with our aim to acquire the images in one single breath-hold. The new sequences used in this study present a good balance between short acquisition time and optimized spatial resolution. This has been achieved in three different ways. Parallel imaging has been the key to reduce the acquisition time especially for the 3D GRE tra sequence using a CAIPIRINHA acceleration technique. Concerning the TRUFI sequence, the echo time has been reduced, at the cost of a very asymmetric echo, resulting in a lack of detail in the frequency direction. Finally, the 3D GRE UTE sequence benefited from in-plane spiral undersampling, without deteriorating the image quality by using an iterative reconstruction SPIRiT.

S1 Table lists all measured diameters of the injected nodules using CT and the three MR-sequences.

### Sensitivity of nodule detection

**Fig 3:**
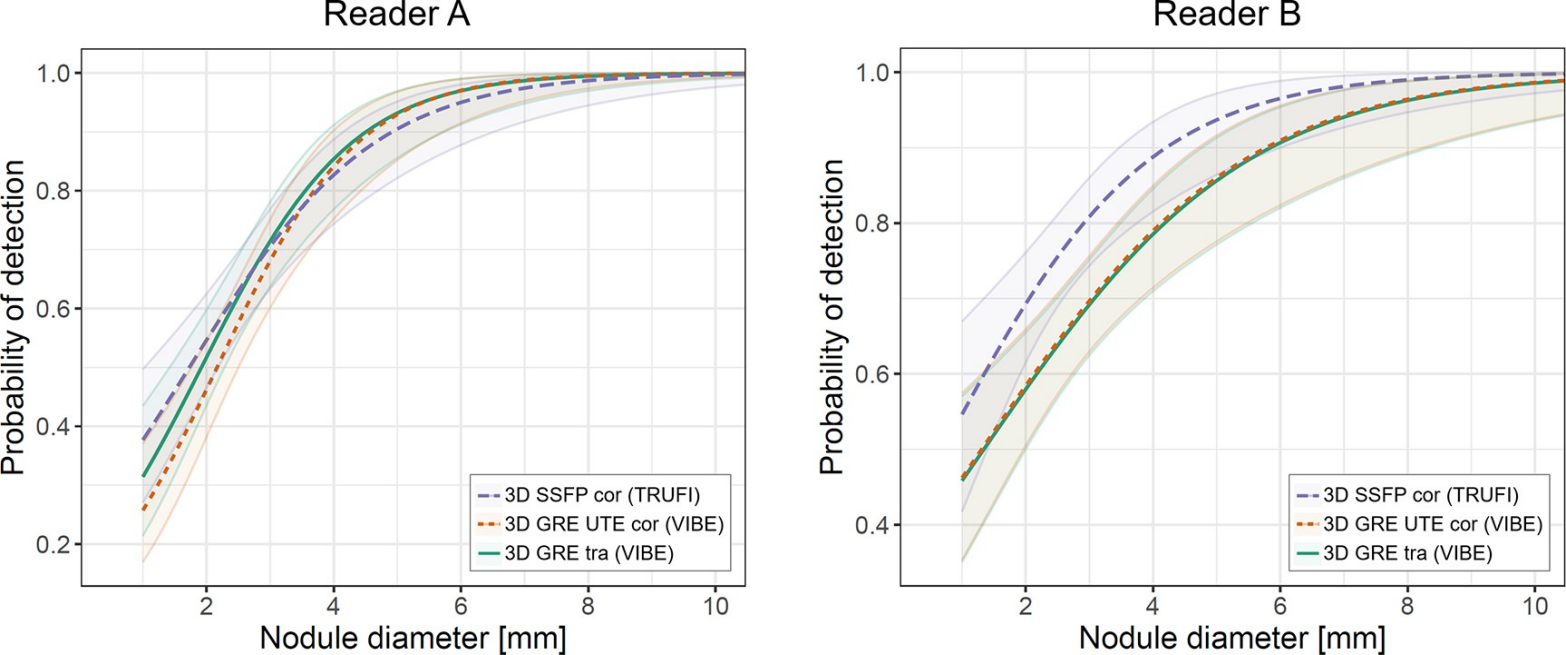
Logistic regression analysis illustrating the estimated probabilities of nodule detection depending on the nodule’s diameter for each MR sequence and each reader. The shaded areas around the lines indicate the 95% confidence interval.

The results of the logistic regression analysis are illustrated in Fig 3. Table 2 resumes sensitivity values for selected nodule diameters according to threshold sizes of the Lung-RADS Assessment Categories [17]. Nodules with a diameter of 8 mm were detected with a sensitivity of 0.99 by Reader A in all three MR sequences. For Reader B, the sensitivity values for detecting 8 mm nodules reached from 0.96 in both VIBE sequences to 0.99 in the TRUFI sequence. Detecting 6 mm lesions was less probable: Reader A reached sensitivity values from 0.97 in both VIBE sequences to 0.95 in the TRUFI sequence; whereas Reader B reached a sensitivity value of 0.96 in the TRUFI sequence, opposed to a sensitivity of 0.91 in both VIBE sequences. For 4 mm nodules the sensitivity values decreased further, dropping under 0.9 as listed in Table 2. Lastly the sensitivity for the detection of 2 mm lesions ranged between 0.46 and 0.69.

**Table 2:** MRI sensitivities (± standard deviation) for different nodule diameters according to logistic regression analysis.

Pulmonary nodule’s diameter (mm)	Reader A			Reader B		
	3D SSFP cor (TRUFI)	3D GRE UTE cor (VIBE)	3D GRE tra (VIBE)	3D SSFP cor (TRUFI)	3D GRE UTE cor (VIBE)	3D GRE tra (VIBE)
**2**	0.55±0.04	0.46±0.04	0.52±0.04	0.69±0.04	0.58±0.04	0.58±0.04
**4**	0.083±0.04	0.84±0.04	0.85±0.04	0.89±0.03	0.79±0.03	0.78±0.04
**6**	0.95±0.03	0.97±0.02	0.97±0.02	0.96±0.02	0.91±0.03	0.91±0.02
**8**	0.99±0.01	0.99±0.01	0.99±0.01	0.99±0.01	0.96±0.02	0.96±0.02

For both readers, the 95% confidence intervals for all nodule sizes overlap, signifying that there is no statistically significant difference between the three tested MR sequences. As this effect is even more distinctive for Reader A, it seems that in the diagram for Reader B the TRUFI sequence shows a trend towards higher sensitivities compared to the other sequences.

Table 3 lists sensitivity values calculated by using selected nodule size intervals to facilitate comparison to Table 2. The results are in agreement with the logistic regression.

**Table 3:** MRI sensitivities by nodule size intervals.

Nodule size intervals (mm)	Reader A			Reader B		
	3D SSFP cor (TRUFI)	3D GRE UTE cor (VIBE)	3D GRE tra (VIBE)	3D SSFP cor (TRUFI)	3D GRE UTE cor (VIBE)	3D GRE tra (VIBE)
**x<3**	0.51	0.44	0.50	0.69	0.55	0.57
**3≤x<5**	0.84	0.81	0.82	0.85	0.82	0.78
**5≤x<7**	1.00	1.00	1.00	0.95	0.95	0.86
**7≤x**	0.97	1.00	1.00	1.00	0.95	1.00

### Positive predictive value

False-positive findings are listed in Table 4. Numerous false-positive findings were identified as occlusions in vessels or bronchi by comparing the MR findings to the CT reference. The PPVs for each MR sequence and each reader are listed in Table 5. Including all false-positive findings leads to low PPVs of less than 0.86. Excluding the artificial false-positive findings identified as vessels or bronchi, greatly improves the PPVs, resulting in values of 0.91 to 0.96.

**Table 4:** False-positive findings for each sequence and reader.

MR-Sequences	Reader A			Reader B		
	Vessels	Bronchi	Other	Vessels	Bronchi	Other
**3D SSFP cor (TRUFI)**	14	25	7	23	27	20
**3D GRE UTE cor (VIBE)**	19	21	10	16	33	8
**3D GRE tra (VIBE)**	12	20	9	7	16	7

**Table 5:** Positive Predictive Value (PPV).

MR-Sequences	PPV including all FP findings		PPV excluding FP findings identified as vessels or bronchi	
	Reader A	Reader B	Reader A	Reader B
**3D SSFP cor (TRUFI)**	0.80	0.74	0.96	0.91
**3D GRE UTE cor (VIBE)**	0.81	0.86	0.95	0.96
**3D GRE tra (VIBE)**	0.77	0.76	0.94	0.96

### Bland Altman analysis

**Fig 4:**
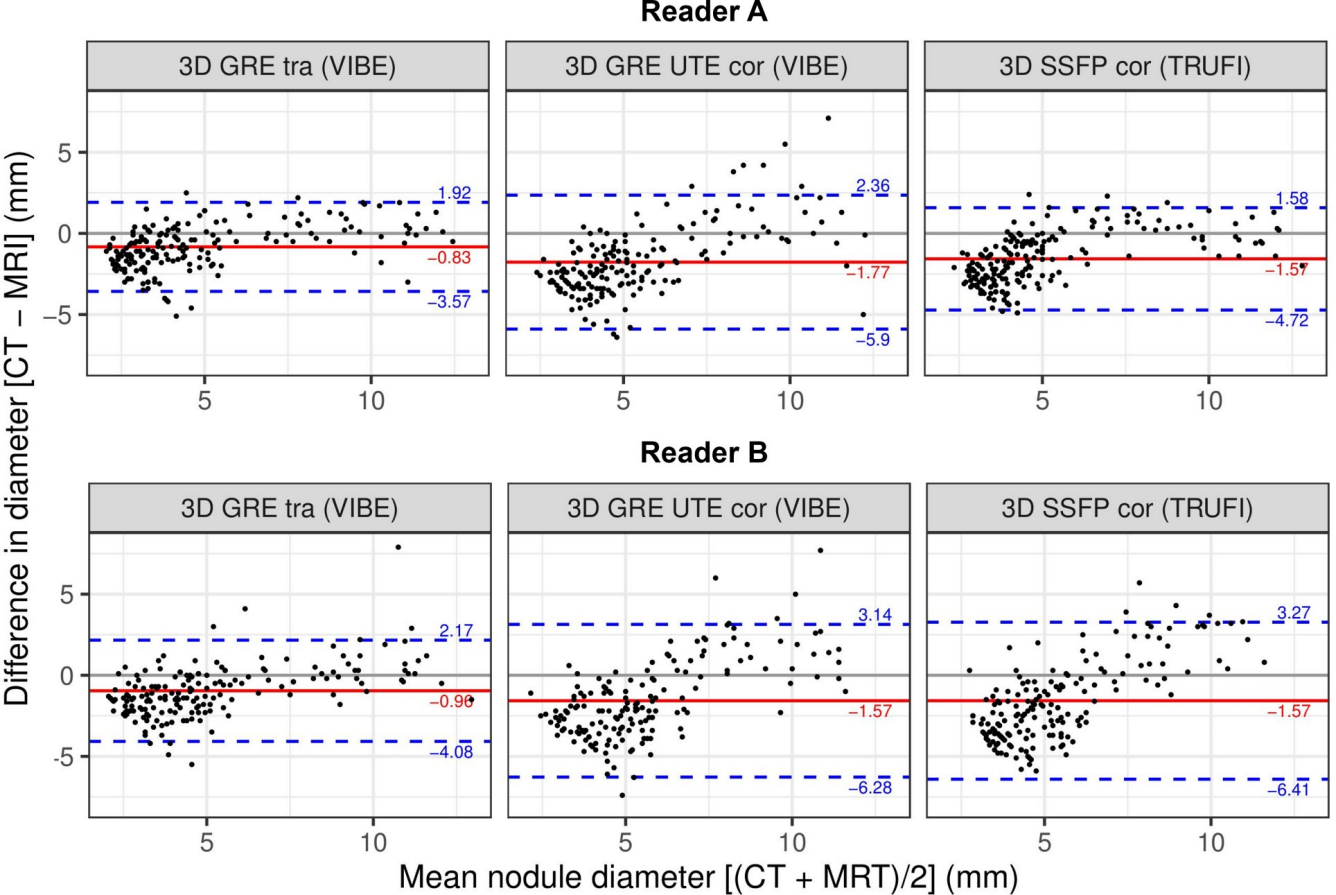
Bland-Altman-plot showing the differences between the nodule size measurements using MRI vs. CT for each of the tested sequences and each reader. The red lines indicate the bias (mean difference between CT and MR measurements), and the dashed blue lines indicate the 95% confidence interval (±1.96 × standard deviation).

To investigate the accuracy of the diameter measurements, Bland-Altman plots are shown in Fig 4 for each Reader separately, illustrating the differences between the MR and the CT measurements as a function of the mean value for the nodules’ diameters.

As visualized by a corresponding line in each Bland-Altman plot, we calculated a bias, indicating the mean difference between CT and MR measurements. For Reader A we found values of -0.83 mm for the transversal VIBE sequence, -1.77 mm for the UTE VIBE sequence, and -1.57 mm for the TRUFI sequence. In this context, the transversal VIBE sequence also showed the best precision, with a standard deviation of 1.4 mm, followed by the TRUFI sequence with 1.6 mm and the coronal UTE VIBE sequence with 2.11 mm. This trend was confirmed by Reader B, yielding the smallest bias of -0.96 mm and smallest standard deviation of 1.59 mm for the transversal VIBE sequence as well.

Furthermore, the following correlation is apparent in all diagrams: the size of small nodules tends to be overestimated in MR images, whereas the size of large nodules is more likely to be underestimated in MR images.

### Interrater reliability

Throughout most of our results, we distinguish between Reader A (2 years of experience) and Reader B (4 years of experience). The interrater reliability for detecting pulmonary nodules in MR images was substantial for both 3D GRE sequences with κ_UTE_ = 0.63 for the coronal VIBE sequence and with κ_VIBE_ = 0.69 for the transversal VIBE sequence. With a κ_TRUFI_ = 0.56, the TRUFI sequence only showed a moderate interrater reliability.

## Discussion

Within this study, we assessed the potential of modern MRI sequences to compete with conventional CT for the detection of pulmonary nodules. In addition to a transversal 3D GRE VIBE sequence, which was found to have a high diagnostic accuracy by Biederer et al. [15], we employed two state-of-the-art pulse sequences, which have shown great potential for lung imaging, namely a SSFP [18] and a UTE [19, 21] sequence. Using a dedicated chest phantom, we were able to simulate a human thorax and repeat examinations across modalities with a perfect reproducibility [15, 23].

In order to mimic a clinical setting, we applied the constraint that all MR sequences could be performed during a single breath-hold of 18 seconds duration. Taking into account the available sequences and techniques, our choice was guided by the aim to obtain a 3D high-resolution isotropic volume within this time constraint. Therefore, gradient echo sequences were preferred, as they are fast and accessible for parallel imaging. In particular, contemporary 3D SSFP sequences allow for shorter repetition times than conventional GRE sequences, facilitating faster k-space coverage and, therefore, lower parallel imaging factors. However, the use of asymmetric short echoes comes at the expense of a possible susceptibility to imaging artifacts. 3D UTE sequences, on the other hand, offer a greatly improved signal to noise ratio in the lung parenchyma, owed to their ultra-short echo times.

All the parameters of the sequences were optimized by a medical physicist.

Logistic regression was used to analyse the probability of detection according to the nodule size for each tested MR sequence. In general, MRI showed a good sensitivity for the detection of large nodules with a diameter ≥ 6 mm, while the probability of detection of smaller nodules decreased rapidly. Similar results have been published earlier [12, 13, 15, 24], stating the inferiority of different MR sequences to CT, especially for lesions smaller than 5–6 mm in diameter. The results of the logistic regression were compared to standard analysis using nodule size categories showing a good agreement between the two analysis methods.

The Lung-RADS Assessment Categories are predicated on the measured diameters of lung nodules with different threshold values for solid lesions at 4 mm, 6 mm, and 8 mm [17]. Additionally, within the framework of the National Lung Screening Trial (NLST) every”non-calcified nodule measuring at least 4 mm in any diameter” detected with low-dose CT, has been considered a positive finding [7]. Both guidelines demonstrate that the reliable detection of pulmonary nodules with a 4 mm diameter is crucial for a lung screening tool. In that regard, we found the tested MR sequences to be lacking sensitivity, as the 4 mm nodules had a probability of detection of less than 0.9. Accordingly, the tested MR sequences are not sensitive enough to be used as screening tool for lung cancer. Recently, Yip et al. [25, 26] re-evaluated the results of the NLST [7]—a study mostly weakened by its high number of false positive findings. They found that higher threshold nodule sizes lead to less cancer diagnosis at the baseline CT scan and prolongate the interval to the next follow-up exam, reducing the number of false-positive findings and the possibility of overtreatment. Considering higher threshold sizes of more than 4 mm, a re-evaluation of MRI as radiation-free option for lung cancer screening would be appropriate.

Comparing ultra-low-dose CT and MRI as possible solutions addressing the problem with the accumulating radiation dose through repeated examinations, we may state the following: MRI has the advantage of being void of ionizing radiation and performs similarly to ultra-low-dose CT in the study of Huber et al. in 2016 [27]. On the other hand, they assert, that the sensitivity of ultra-low-dose CT can be enhanced by additional processing such as CAD software [27].

To facilitate a correct nodule categorization according to the Lung-RADS criteria and to assess its growth precisely throughout repeated examinations, an accurate size measurement of the nodule is crucial. Using the three tested MR-sequences, small nodules were more likely to be overestimated in size, whereas the size of larger nodules was rather underestimated. The transition point ranges between 5 and 7 mm nodule diameter, depending on the applied sequence and reader. Due to this relationship, it is difficult to interpret the accuracy of the size measurements. However, the average MRI-determined nodule size shows a good congruence to the CT measurements, with a bias of -0.83 mm to -1.77 mm. These findings are in line with a study by Cieszanowski et al. [14], who found a very strong correlation between the CT and MR measurements with differences ranging between -1.6 mm and +1.57 mm. Partial-volume effects are likely to cause the overestimation of small nodules. A possible explanation for the underestimation of large nodules could be as follows: As large nodules—with a diameter > 7 mm—are facile to detect, the reader is less likely to use strong contrast settings. Consequently, the expansion of large lesions might not be captured fully, leading to a biased size measurement.

Being not yet suitable as a lung cancer screening tool, MRI may figure as imaging modality for some follow-up examinations, presupposed the monitored lung lesions have a diameter ≥ 6 mm. The “Fleischner Criteria” serve as guidelines for incidental pulmonary findings in CT scans, proclaiming that a follow-up exam is needed, if the pulmonary lesion displays a diameter ≥ 6 mm, if it portrays some malignancy criteria (in high-risk patients) or if there are multiple nodules [28]. In those cases of nodules with diameter ≥ 6 mm, MRI could be considered as follow-up examination tool in order to reduce the cumulative radiation dose for those patients.

The diagnostic precision—expressed by the PPV—was strongly impaired by false-positive findings, most of which were identified as obstructed vessels or bronchi considering the CT images as reference, resulting in PPV values between 0.74 and 0.86. Compared to *in vivo* MR images of the lungs, occluded vessels and bronchi were more likely to be confounded with lung nodules, due to our study set-up. Therefore, we opted to exclude subsequently those specific false-positive findings from the PPV calculations. After this correcting step as depicted in Table 5, both readers reached PPV values of more than 0.9 for all MR sequences. Accordingly, the tested MR sequences detect pulmonary nodules with a promising precision with a small remaining risk of a false positive diagnosis.

The calculated, unweighted kappa showed substantial interrater reliability for both VIBE sequences and moderate interrater reliability for the TRUFI sequence. Even though the latter sequence displays a good spatial resolution, it was particularly impacted by artifacts, most likely related to the asymmetric echoes. These artifacts have probably bothered one reader, leading to a poorer performance analysing the TRUFI MR images.

In summary, since all three tested MR sequences performed similar throughout the experiment in terms of sensitivity and nodule size measurements, there is little evidence for the superiority of modern MR sequences, such as the 3D GRE UTE, for nodule detection. Nonetheless, the short echo-times of the UTE sequence may have advantages in the detection and characterization of non-nodular changes in lung tissue which was not the focus of this study. The TRUFI sequence seems less eligible for screening purposes, because of its moderate interrater reliability. While the echo time has been reduced to limit magnetic susceptibility artifacts, other image artifacts remain due to the usage of asymmetric echoes. Hence, a combination with parallel imaging could help resolve this problem, although the possibilities for 3D parallel imaging on this particular sequence are still limited. Once these limitations are addressed, a TRUFI sequence could play a major role in an MR-based lung cancer screening.

This study has some limitations. Without true negative findings (due to the study design), it was not possible to assess the specificity of the tested MRI sequences. Furthermore, while we were able to inflate the porcine lungs in a physiological manner, it was not possible to simulate cardiac or residual respiratory movements. This lack of motion artefacts may have enhanced the established sensitivity values. Conversely, the absence of perfusion of the heart-lung preparations generally caused a poorer image quality compared to MR images of living patients, impeding the detection especially of smaller nodules.

As this study is meant to elucidate the sensitivity of MRI, we did not dwell on the CT performance of detecting suspect lung lesions–a subject elaborately discussed in the literature [29].

Lastly, using the agar—prune juice mixture (cf. simulation of the lung nodules) we emulated the relaxation times of real pulmonary nodules, but we must take into account that lung lesions can still show varying MRI signal intensity and can adopt different shapes and appearances (solid, part-solid, translucent, etc.), as they originate from various cells and tissues. This applies in particular to the appearance of ground glass nodules, which have not been considered in this study.

In conclusion, assessing the optimized MR sequences showed the capabilities of MRI as a radiation-free imaging modality, but referred also to its remaining inadequacies: nodules with 6 mm in diameter and larger could be detected with a high sensitivity; however, for smaller nodules the sensitivity declines rapidly. Accordingly, MRI is not yet suitable for lung cancer screening. Nonetheless, the three tested MR sequences yielded high positive predictive values and could potentially be used as follow-up examination tool in selected scenarios. Finally, as considering larger threshold sizes for nodule categorization has already been discussed in the literature [25, 26], MRI may serve as a radiation-free lung cancer screening tool in the future.

## Supporting information

S1 Table Diameter of all injected nodules as measured using CT and the tested MR-sequences. (XLSX)
